# Supplementary material for: Are carbon emissions trading and green financial instruments synergistic? -Comprehensive quantitative research based on content analysis
Source: PLoS One. 2024 Mar 7;19(3):e0298601. doi: 10.1371/journal.pone.0298601 (PMC10919601; doi:10.1371/journal.pone.0298601)
Supplement: S1 Data — (ZIP) [file pone.0298601.s001.zip › Data/地级市Z_F工作报告.zip!/╣π╬≈/╙±┴╓╩╨/└┤╫╘╣1⁄2╓┌║┼í╛┬φ┐╦╩2╛▌═°í┐_2015.docx]

各位代表：

现在，我代表市人民政府向大会作工作报告，请予审议，并请市政协委员和列席会议的同志提出意见。

一、2014年工作回顾

2014年是不平凡的一年，面对复杂严峻的形势和艰巨繁重的改革发展稳定任务，在自治区党委、政府和市委的领导下，我们主动适应经济发展新常态，牢牢把握稳中求进的工作总基调，以开展“三个年一活动”为总抓手，深入实施“三年打基础，五年大变化”、建设“两城市一中心”战略部署，加快“三个全面”，推进“三个转变”，从容应对挑战，奋力攻坚克难，实现了经济社会持续健康发展。

——经济运行稳中有进。完成地区生产总值1341.7亿元，增长8.4%；财政收入128.2亿元，增长12.6%；固定资产投资1123.7亿元，增长17.8%；社会消费品零售总额545.7亿元，增长13%；规模以上工业增加值410.9亿元，增长11.3%；外贸进出口总额4.9亿美元，增长16.9%。财政收入、社会消费品零售总额等6项主要经济指标增幅排全区前3位。

——民生质量不断提高。全市财政民生支出173.8 亿元，增长9.8%，占财政支出的比重为75.8%。农民人均纯收入9314元，增长12.6%；城镇居民人均可支配收入26681元，增长9.5%。城乡居民收入差距进一步缩小。

——重大项目实现突破。50万吨镍合金项目一期建成投产；广西“五彩田园”现代特色农业示范区开园；再生资源园区获选为第五批国家“城市矿产”示范基地；玉铁铁路即将通车；黎湛铁路电气化改造和玉林火车站改造实现开工。

——发展活力持续增强。非公经济主体突破15万户，注册资本总量突破600亿元。民间投资超950亿元，占固定资产投资85%以上。五个县（市、区）、玉东新区的财政收入超10亿元。我市获批为全国第二批农村改革试验区，列入国家第一批生态文明先行示范区建设名单，荣获“中国南方药都”称号。

一年来，我们主要抓了以下工作：

（一）抓产业稳增长。安排资金3.2亿元，深入开展“园区建设攻坚年”活动，龙潭产业园、玉柴产业新城、中医药健康产业园等八大园区完成工业投资200亿元，机械制造、有色金属、医药食品等八大产业对工业经济增长的贡献率达60.2%。新增亿元企业31家、高新技术企业3家。实施“3个100示范工程”，“五彩田园”示范区建设稳步推进，全市农业重点龙头企业达161家，农民合作社2464个，家庭农场141个。粮食总产188万吨，中药材种植面积35万亩，水果、蔬菜、养殖、林下经济稳步增长。毅德二期、中药港二期、汽车城等16个重点商贸项目加快推进，阿里巴巴·玉林产业带启动运营，中国特产网总部落户玉林。实施重大旅游项目15个，总投资360.6亿元；实现旅游总收入150.1亿元，增长27.4%，增幅居全区前列。

（二）抓项目强基础。深入推进“征地、打违、处遗”专项行动，完成征地3.5万亩，市本级清理整治“两违”213万平方米，59个安置区规划建设稳步推进。多渠道筹集项目建设资金，为320家中小企业担保融资77亿元，金融机构新增贷款100.5亿元，社会融资总量237亿元。推进480个重大项目建设，完成投资451.9亿元，新开工玉柴气体发动机生产基地二期、西气东输二线接气、国电六坪顶风电、红星国际广场等198个重大项目，玉柴新能源发动机、明旺5万吨复元乳、新盈邦陶瓷、利维重工等96个重大项目竣工投产，发展后劲不断增强。

（三）抓城建提形象。深入开展“城市形象提升年”活动，投资70亿元，实施285个市政项目，建成市博物馆、文化艺术中心、迎宾大道三期、江岸路西段、新民路北段等设施。玉东新区发展步伐加快，玉北同城化稳步推进。加快创建国家园林城市，完成主要城市道路绿化提升、风貌改造和灯光工程，清理户外广告3万平方米，“五乱”现象和“四车”整治效果明显。抓好保障性住房建设，新开工5955套、建成5406套、入住5525套，棚户区改造3842套。实现100%乡改镇，新建社区32个，新增城镇人口3.5万人，城镇化率提高到46%。

（四）抓改革添活力。深化农村综合改革，528个村（社区）农村“六权”确权颁证有力开展，全市农村集体土地所有权、建设用地使用权、林权发证率均达94%以上。成立广西首家市级农村产权交易中心和农村土地流转服务中心，新增农村土地流转27万亩，流转率达52.3%。全面启动工商登记制度改革，新增各类市场主体1.8万户，新增注册资本231.7亿元，增长4倍多。推动企业改制上市，广西汽牛公司等6家企业在新三板、上海股权交易中心和深圳前海股权交易中心挂牌。深化国有企业改革，完成玉柴通用机械公司、市粮油食品进出口公司等企业改制，成立玉柴产业新城投资发展公司和市交通旅游投资集团公司。

（五）抓开放扩合作。加快“东靠南下、通江达海”开放合作步伐，“修机场、建码头、接高铁”三件大事取得突破性进展，民用机场选址获中国民航局批复、项目预可研通过评估，机场公路、铁山港东岸码头纳入自治区统筹推进重大项目，绣江复航工程项目完成可研编制。郁江引水等10个项目列入西江经济带基础设施建设大会战项目。成功举办第六届药博会、第四届陶博会，荣获“2014年度全国优秀会展城市”称号，引进深圳华侨城、康美药业、华夏本草等项目444个。实施加工贸易倍增计划，外贸进出口扭转持续21个月负增长的局面。

（六）抓生态美城乡。深入开展“美丽玉林·清洁乡村”活动，全面启动生态乡村建设，完成103个农村环境连片整治项目，新建农村污水管网207公里、垃圾处理设施459套、沼气池1.9万座，造林绿化27万亩，城乡生态环境明显改善。加强九洲江综合治理，筹集资金6.3亿元，拆除禁养区养猪场795家，开工建设15个污染整治项目，九洲江水质稳步好转。强化能耗监管，万元生产总值能耗下降3.1%。严格控制重点污染物排放，完成污染减排项目983个。全市环境质量总体良好，主要区域空气质量保持国家二级标准，主要饮用水源保持Ⅱ类水质。

（七）抓民生促和谐。投资60.7亿元的11项为民办实事工程全面完成。新建学校16所，玉高新校区、万秀小学等5所学校建成投入使用，新增学位1.1万个。建成村级公共文化服务中心165个，创建国家公共文化服务体系示范区工作通过中期评估。成功举办第三届运动会，全民健身活动深入开展。实施11类44项国家基本公共卫生服务项目，人口计生工作健康发展。新增农村劳动力转移就业5.2万人，新增城镇就业3.3万人。城镇职工、城镇居民和新农合参保率稳定在97%以上。价格总水平保持平稳。完成6个社区规范化建设试点和204个农村幸福院建设。建成78个水库移民新村。259个贫困村扶贫开发稳步推进。在全区率先建成市县镇三级一体民生资金监管服务平台，监管民生资金49.8亿元。严厉打击各种违法犯罪活动，立案数下降15.6%。加强矛盾纠纷排查调处，严格食品药品监管，深入开展安全生产“六打六治”专项行动，和谐稳定局面进一步巩固。

（八）抓作风优服务。深入开展党的群众路线教育实践活动，聚焦“四风”问题抓好整改，健全作风建设长效机制。取消行政审批项目76项、下放36项，精简率达13%。减少会议和文件20%以上，公务用车购置和运行费用、公务接待费用、因公出国（境）费用支出同比分别下降12.1%、43.3%、65.8%。认真履行党风廉政建设“两个责任”，贯彻落实“八项规定”和“约法三章”精神，严肃查处违法违纪问题。坚决贯彻执行市委的决策部署，认真执行市人大及其常委会的决议、决定，自觉接受人大、政协和社会监督，认真听取各民主党派的意见，人大代表建议和政协委员提案全部办结。行政监察和审计监督进一步加强。政务公开和信息公开不断深化。

国防教育和国防后备力量建设不断加强，驻玉部队在支持地方建设上做出重要贡献，军政军民团结局面进一步巩固，实现再创自治区双拥模范城。中直、区直驻玉各单位为经济社会发展提供大力支持。民族、宗教、外事、侨务、对台事务、人防、统计、供销、地震、气象、水文、无线电管理、农机、方志、档案、机构编制、发展研究、住房公积金管理、老龄、残疾人、科普等工作取得新成绩。工会、共青团、妇联等群团组织重要作用得到进一步发挥。

各位代表，过去一年的成绩来之不易。这是自治区党委、政府和市委坚强领导的结果，是市人大依法监督的结果，是市政协和社会各界监督支持的结果，是全市人民团结奋斗的结果。在此，我代表市人民政府，向全市人民，向人大代表、政协委员，向中央及自治区直属驻玉单位、驻玉部队、武警官兵，向各民主党派、工商联、无党派人士、各人民团体和海内外所有关心、支持玉林发展的各界人士，表示崇高的敬意和衷心的感谢！

在肯定成绩的同时，我们也清醒地看到发展中存在的困难和问题。在严峻的经济形势下，尽管我们采取了一系列积极措施，但我市经济下行压力持续加大，企业发展困难加剧，经济增长速度放缓；基础设施不完善，要素瓶颈制约突出，发展后劲不足；传统产业优势弱化，新兴产业培育发展不快，转方式调结构任务艰巨；保障和改善民生的压力较大，社会事业发展滞后；行政效能需要进一步提升，投资发展环境有待改善，等等。对此，我们将高度重视，认真研究，务实解决。

二、2015年工作安排

今年是“十二五”规划的收官之年，是全面推进玉林法治建设的开局之年，是推进“三年打基础，五年大变化”的攻坚之年。我国经济发展进入新常态，国内外发展环境依然错综复杂，经济持续下行的压力依然很大，我们既面临经济增速减挡带来的影响，也面临经济结构调整、发展方式转变、发展动力转换蕴藏的机遇。中央坚持稳中求进，全面深化改革，推进创新驱动，支持中西部地区基础设施建设，支持广西打造“一带一路”有机衔接的重要门户，支持北部湾经济区和珠江—西江经济带建设，一系列稳增长措施的政策效应将进一步显现，这些都将为我们拓展了更为广阔的发展空间。在经济发展新常态下，我们要把玉林放到全区乃至全国的大背景下找准定位、发挥优势、精准发力。既要增强忧患意识，立足当前，保持经济平稳较快增长；更要增强机遇意识，着眼长远，着力提高发展质量，在创新中转型、在转变中发展。

今年政府工作的总体要求是：深入贯彻党的十八大和十八届三中、四中全会精神和习近平总书记系列重要讲话精神，主动适应经济发展新常态，坚持稳中求进工作总基调，以提高经济发展质量和效益为中心，坚持总量提质、分量提速，以转方式调结构为重点，以“三个年一活动”为总抓手，深入推进“三年打基础，五年大变化”战略部署，深入推进“东靠南下、通江达海”战略举措，深入推进“两城市一中心”建设，突出创新驱动，深化改革开放，注重生态文明，全力改善民生，促进经济健康较快发展和社会和谐稳定，为与全区全国同步全面建成小康社会奠定坚实的基础。

今年经济社会发展的主要预期目标是：地区生产总值增长8.5%；财政收入增长8%；规模以上工业增加值增长11.5%；固定资产投资增长16%；社会消费品零售总额增长12%；外贸进出口总额增长12%；城镇居民人均可支配收入增长9%；农民人均纯收入增长11%；人口自然增长率、城镇登记失业率、节能减排等指标控制在自治区调控目标以内。

确定上述目标，主要是从适应新常态、实现“十二五”规划和全面建成小康社会目标，以及资源环境支撑等综合因素考虑。把经济预期增速适当调下来，把体现质量和效益的目标提上去，着力转型升级，创新驱动，补齐发展短板，挖掘和培育新的增长点，在采取有力措施稳增长的同时，积极推进调结构转方式，努力增强发展的动力和后劲。为此，我们将重点抓好以下工作：

（一）着力创新驱动，在加快工业转型升级上奋力突破。

大力提高创新能力。加快玉林高新区、清华科技园建设，积极引进一批高新技术企业，打造创新集聚区。建设市创业孵化基地、玉柴工业园孵化中心、高新区孵化中心，完善创新服务平台。建设完善高效节能环保内燃机国家工程实验室、国家级陶瓷重点实验室，积极申报国家火炬计划内燃机特色产业基地，发挥创新引领作用。开展智慧城市试点创建工作，推进云计算、大数据、物联网与制造业深度融合，促进产业创新发展。支持企业建立研发机构，与科研院所共建研发基地，进行技术、产品、管理、业态等方面的创新，培育一批科技型企业。

大力推进园区建设。安排资金2.9亿元，支持八大园区、八大产业发展。加快玉柴产业新城建设，开工建设中原内配、银轮机械、柴油机装备制造等项目。加快龙潭产业园中金镍合金及深加工二期、银亿技改等项目建设，确保再生资源园区通过国家验收。加快中医药健康产业园建设，抓好康美中药加工、玉药中药提取基地、中国（玉林）—文莱中医药健康产业生产基地等项目。统筹推进市经济开发区、北流日用陶瓷产业园等园区建设发展。创新园区管理机制，最大限度授予园区经济管理权限，增强园区发展活力。力争完成园区固定资产投资278亿元，建成标准厂房150万平方米，实现工业总产值1745亿元。

大力优化工业结构。以重大项目促进结构调整，做优存量，做大增量。围绕八大产业和特色产业，实施亿元以上重大工业项目172个，力争竣工55个，重点抓好42个投资5亿元以上的项目，推动机械制造、有色金属、陶瓷等传统产业转型升级，促进再生资源、新能源新材料、节能环保等战略性新兴产业发展壮大。积极发展铁锅、芒编、农机、服装、皮件等县域特色产业，增强县域经济活力。

大力支持企业发展。加快企业融资平台建设，支持企业开展技术改造，促进“个转企”“小上规”“规上市”，力争亿元企业达到295家，培植上市后备企业30家以上。加强企业自主品牌建设，扩大市长质量奖范围，支持企业争创驰名商标、名牌产品，增强企业竞争力。

（二）着力现代服务业发展，在建设北部湾城市群商贸中心上奋力突破。

积极发展旅游业。把旅游业作为重点推进的八大产业之一，加大发展力度，积极创建国家健康旅游示范市。加快“五彩田园”、佛子山、都峤山、大容山、真武阁、鹿峰山、六万大山等重点项目建设，力争两到三年新增国家5A级景区1个、4A级景区4个、四星级以上乡村旅游区3个、四星级以上农家乐10家，新增五星级酒店2家、四星级酒店5家。支持容县、北流、陆川创建广西特色旅游名县。做好中泰（玉林）旅游文化产业园落户工作，积极申报世界自然遗产和文化遗产。规划建设玉林文化产业商贸城和中国—东盟创意印刷产业园，促进文化与旅游融合发展。

积极发展商贸物流业。打响“中国南方药都”品牌，加快中药港二期等项目建设，力争组建中国—东盟（玉林）中药材交易所，积极发展中药材电商、远期交易、仓储物流、生物制药、健康旅游等现代先进业态，力争通过3~5年的努力，把玉林建设成为中国—东盟自由贸易区最大的中药材集散地、交易中心、定价中心和中国医药健康国际名城。加快推进毅德二期、宏进二期、红星美凯龙等重大项目建设，促进现代商贸物流业集聚发展。完善商业网点规划，推进大型商业街、购物中心等综合体建设，抓好10个农贸市场建设改造，发展提升传统商贸业。加快金城商厦综合体建设，打造10亿元商业龙头企业。

积极培育新型服务业态。大力培育发展电子商务、科技服务、文化与创意、软件与信息服务、物业管理等新兴服务业。推动专业市场大数据采集体系建设，在城区1~2家专业市场实行统一电子结算。加快市电子商务快递物流园、阿里巴巴·玉林产业带等电子商务平台建设，积极发展行业电商、农村电商、社区电商，培育1家年交易额超10亿元的电商企业。促进快递产业与制造业、农业、电子商务联动发展。培育2~3家国家级物业公司。

积极扩大城乡消费。落实国家收入分配政策，积极推进城乡居民就业创业，持续增加居民收入，提高居民消费能力。继续办好“岭南都会”消费购物节，支持举办房产汽车、家居建材、农林产品等专业性展销会、交易会，活跃城乡市场。

（三）着力转变发展方式，在发展现代农业上奋力突破。

加快“五彩田园”现代特色农业示范区建设。加快中国杂交水稻援外实训基地、海试区科技园、中国南药园等重大项目建设，大力发展特色农业、生态农业、观光农业、农产品加工业，加快生态田园建设和村庄风貌改造，努力建成农业科技展示、中草药种植加工、农耕文化教育、职业农民培训以及乡村休闲旅游“五位一体”的现代特色农业示范区，加快建设广西五星级乡村旅游区和国家5A级景区，打造绿色生态、田园都市品牌。

加快发展优势特色产业。扩大粮食、蔬菜、水果种植规模，力争粮食总产184万吨以上，蔬菜种植155万亩，水果230万亩，推进粮食生产现代化，打造中国南方农谷。积极发展中药材种植、花卉产业和林下经济，力争中药材种植40万亩以上。推进养殖业转型升级、提质发展。鼓励农业企业和合作组织争创无公害农产品、绿色食品、有机食品、农产品地理标志，打造一批农业品牌。积极发展农产品加工业，促进农业向二、三产业延伸。

加快培育新型经营主体。实施创建100个现代特色农业示范区、100个农民合作示范社、100家示范性家庭农场的“3个100示范工程”，引导农民以土地承包经营权入股农民合作社和农业企业，促进农业规模经营、集约发展。强化农业科技创新驱动作用，支持农业企业、农民合作社、家庭农场等经营主体引入科技和现代经营手段推进现代农业建设，提高农产品加工率和商品率，增强市场竞争力。

加快农民增收步伐。继续实施农民收入倍增计划。鼓励农民优化种养结构，提高家庭经营性收入。发展面向农村的职业教育，提高农民职业技能，增加农民工资性收入。积极发展集体经济、合作经济，扩大农民土地、林权收益，增加农民财产性收入。完善农业补贴制度，发展政策性农业保险，增加农民转移性收入。

（四）着力重大项目建设，在夯实发展基础上奋力突破。

努力扩大投资规模。积极开辟投资新领域，扩大有效投资，发挥投资对稳增长调结构转方式的关键性作用。实施亿元以上重大项目550个，年度完成投资515亿元，其中新开工项目189个，续建项目193个，竣工项目31个，开展前期工作项目137个。力争列入自治区统筹推进重大项目30个以上。

加快重大项目建设。开工建设华电热电联产、恒科药业异地搬迁改造、中广核风电、路宝水泥技改等项目。力争玉柴铸造中心三期、气体发动机、郁江引水及城北水厂扩建工程等项目竣工投产。加快推进临空产业园、玉林无水港、北流华润风电、六万山风电等一批重大项目前期工作。

做好重大项目储备。围绕构建完善的基础设施体系、现代产业体系、公共服务体系、生态支撑体系，组织编制好“十三五”规划，深入研究和谋划一批打基础、利长远、增后劲的重大项目、重大工程，力争列入广西“十三五”规划重大项目370项以上。

努力突破瓶颈制约。严格土地管理和耕地保护，用好国家土地政策，深入开展“征地、打违、处遗”工作，推进土地市场化开发，节约集约用地，保障项目建设用地。全面加快59个安置区建设。多渠道争取项目资金，力争新增贷款100亿元，完成民间投资1000亿元。

（五）着力城市扩容提质，在建设区域性大城市上奋力突破。

做大中心城区。完善城市规划体系，强化玉林区域性中心城市地位。大力推进玉东新区建设发展，统筹加快基础设施、产业、生态和公共事业项目建设，促进与北流城区发展对接，加快玉北同城化步伐，拓展新一轮城市发展框架。抓好火车站站房改造、玉北大道、胜利路、文体路等一批市政项目建设，完善城市设施，提升城市功能。大力推进现代城市综合体建设，加快红星国际广场、富林·汇金广场等项目建设，力争开工建设大连万达、步步高、福绵香港新城等项目，提升城市品位。

做优城市环境。抓好城市9大主题公园、南流江百里景观长廊等项目建设，继续实施小游园、小广场等“五小”工程，推进城区主干道及小街小巷绿化建设，加快创建国家园林城市。积极申办广西园博会。抓好教育东路、江滨路、民主南路等主干道风貌改造。积极发展公共交通、管道燃气、供水供电、信息通讯等公共事业，推进“市民卡”建设工程。继续深化“五乱”整治和“四车”治理，推进规范管理向城中村、城乡结合部、乡村延伸。

做强县城和小城镇。实施大县城战略，加快基础设施建设，增强城区承载能力。继续推进博白等县的撤县改市工作。抓好重点镇和中心镇建设，努力建设一批经济强镇。积极推进镇改街、村改社区，深入开展社区规范化建设攻坚活动，建成50个规范化社区，力争我市社区信息综合服务平台建设列入国家惠民试点工程。坚持人口城镇化核心，促进农业转移人口市民化，年内全市城镇化率达到47.5%。

做实棚户区改造。把棚户区改造作为加快城镇化和改善民生的重要措施，抓好火车站片区等25个棚户区改造项目，改造棚户区7309户。投资1.1亿元，建设公共租赁住房1478套。

（六）着力深化改革，在建设国家非公经济发展示范城市上奋力突破。

深化农村综合改革。落实全国第二批农村改革试验区建设试点任务，加快建设覆盖全市、城乡统一的农村产权流转交易市场体系，设立市县农村产权流转交易风险补偿基金，推动农村经营性集体建设用地、农村土地承包经营权、林权等各类农村产权入市流转交易。稳步发展农村土地股份合作、土地信托等新型经营模式，推进农村土地向科技人员、种田能手、专业大户、农民合作社和龙头企业流转，激发农业和农村发展活力。

深化投融资体制改革。进一步减少投资审批事项，规范投资审批行为。积极开展股权融资、产权交易融资、债券融资等多样化融资。探索引入风险投资。扩大民间资本市场准入范围，鼓励和吸引民间资本以合资、独资、特许经营等方式参与项目建设运营，拿出一批基础设施和公共事业项目实行ppp融资试点。深化价格改革，放开一批商品和服务价格。

深化农村金融改革。加快推进农村信用社改组改制成农村商业银行，推动村镇银行分支机构向乡镇农村基层延伸。加大农村金融产品创新力度，推动保险、信托、基金、股权、债券等各类金融产品进入“三农”领域。加快农村信用体系建设，构建金融风险防范化解机制。

深化全民创业。厚植创业创新文化，营造创业创新氛围，推进全民创业万众创新，全力推动非公有制经济发展壮大。深化工商登记制度改革，降低市场准入门槛，优化准入流程，大力培育各类市场主体。完善创业引导基金、优惠贷款、技术服务等政策制度，帮助中小企业度过初创期，步入成长期，迎来收获期。

（七）着力开放合作，在拓宽发展空间上奋力突破。

加快交通枢纽建设。继续抓好“修机场、建码头、接高铁”三件大事，主动对接国家主路网、主骨架，构建航空、港口、铁路、公路互联互通的现代综合交通体系。航空方面，开工建设玉林民用机场及机场大道。港口方面，开工建设铁山港东岸码头，加快推进绣江复航前期工作。铁路方面，加快黎湛铁路电气化改造步伐；力争开工洛湛铁路梧州至玉林段电气化改造，做好玉林至铁山港铁路复线电气化前期工作，争取张家界经玉林至海口高铁列入国家“十三五”规划。高速公路方面，开工建设荔浦至玉林高速公路，加快南宁苏圩至北流清湾、松旺至铁山港高速公路前期工作，争取玉林至湛江高速公路列入国家规划。公路路网方面，加快龙潭产业园至铁山港东岸一级公路、岑玉线北流甘村至新圩段改造前期工作，做好玉林快速环路、马坡至盘龙、石和至博白等一级公路规划，推进岑溪南渡至陆川、贵港木格至博白水鸣、陆川清湖至浦北石埇等二级公路建设。

提高开放合作水平。主动融入“一带一路”战略，参与东盟及其他国家的交流与合作。深化与珠三角、港澳台、西南、中南地区的合作，抓好粤桂合作产业园规划建设。全面融入自治区“双核驱动”战略和“三区统筹”格局，加快龙港新城规划建设，积极参与北部湾经济区通关一体化，抓好西江经济带基础设施建设大会战项目建设，扩大区域合作发展成效。

抓好招商引资工作。办好玉博会、药博会和陶博会，打响玉商品牌。继续实施招大引强，围绕八大产业和县域优势产业，积极开展产业招商、以商引商。加强与央企、民企、外企的招商对接，着力引进一批世界500强、国内500强、民营500强、行业100强企业，力争招商引资到位资金突破500亿元。

发展提升外向型经济。培植和壮大进出口龙头企业，培育进出口额3000万美元以上企业5家、1000万美元以上企业12家。稳定日用陶瓷、编织品、服装等传统优势产品出口，扩大工程机械、高新技术产品等高附加值产品出口，保障重要资源和关键技术设备及零部件进口。深入实施加工贸易倍增计划，加快企业保税仓库建设，实现加工贸易额1.6亿美元以上。

（八）着力生态文明，在建设美丽玉林上奋力突破。

推进生态乡村建设。深入开展“村屯绿化”“饮水净化”“道路硬化”活动，推进植树造林、生态农业、农田水利、村屯道路等项目建设，全面提升农村生态环境。加快推进“五彩田园”生态示范区、玉（林）容（县）一级公路苗木花卉生态产业示范带、玉（林）兴（业）现代农业特色示范带、南流江百里景观长廊、北流河（圭江绣江）旅游观光带、九洲江生态文明示范带建设，努力构建“一区两路三江”生态体系新格局。

强化节能减排工作。严格贯彻实施新《环境保护法》，全面完成“十二五”节能减排任务。加强能耗监管，积极淘汰落后产能。加强公共机构节能工作。加快市污水处理厂二期、污水管网、中水回用、餐厨垃圾处理、建筑垃圾消纳场等设施建设。抓好机动车排气污染监测，加快淘汰黄标车及老旧车。推进城区空气自动站建设和PM2.5监测。大力发展生态产业和循环经济，加快“城市矿产”示范基地建设，争创国家再生资源回收利用体系建设试点城市。

推进九洲江综合整治。加快污水处理厂、垃圾填埋场、垃圾中转站、大中型沼气池等重点项目建设，深入整治养殖污染。全面清除河道污染物，形成河道清理长效机制。推进沿岸涉水企业转型升级，引导鼓励企业入园发展。大力推进造林绿化，增强水源涵养能力，构建水生态安全保障体系。

（九）着力民生保障，在提高人民生活水平上奋力突破。

抓好为民办实事工程。多渠道筹措资金，实施社保惠民、健康惠民、教育惠民、强基惠民、土地整治惠民、安居惠民、农补惠民、生态惠民、文化惠民、交通惠民等10大民生工程项目，重点推进农村公路、农村居民安全饮水、农户住房保险、城镇保障性住房、幼儿园、绿化示范村屯等六个方面的民生项目。充分发挥民生资金监管服务平台作用，强化民生资金监管，确保人民群众得到更多实惠。

做好就业和社会保障工作。完善公共投资促进就业机制，加强就业服务，支持农民工回乡创业，提高高校毕业生和农民工就业率。实施全民参保登记计划，做好社保扩面征缴工作。推进“五险合一”信息系统升级改造，加快异地就医即时结算平台建设。完善社会救助和防灾减灾体系。加强价格监管，稳定价格总水平。

扎实推进精准扶贫。创新扶贫体制机制，推进扶贫责任、权力、资金、任务“四到县”，加大产业、教育、科技扶贫力度。安排资金1.2亿元以上，实施整村推进扶贫，落实到户、精准到人，实现扶持贫困人口15万人以上，脱贫6万人以上。

提升教育发展水平。筹措资金20亿元，建设中小学（幼儿园）29所，改扩建一批中小学校，促进城乡教育均衡发展。继续实施城区中小学校建设大会战，加快玉林师院附中新校区、玉州区金港小学、玉柴新区小学等学校建设步伐。推进教育改革创新“八大工程”，促进各级各类教育健康发展。加快玉柴职业技术学院、市职业教育园区建设，大力支持玉林师范学院发展，启动广西医科大学玉林校区建设，提升高等教育发展水平。

加强人才队伍建设。深化“人才强市”战略，完善人才培养、引进、选用机制和激励机制，优化人才发展环境。加强人才小高地建设，完善特聘专家等人才制度，培育和引进一批高端人才。积极推行“项目+人才”模式，实现项目、人才双引进。统筹抓好各类人才队伍建设，全面壮大人才队伍。

加快发展文化体育事业。大力创建国家公共文化服务体系示范区，确保如期通过验收。加快市图书馆新馆、王力博物馆、市档案馆新馆等文化设施建设，做好市群众艺术馆、非物质文化遗产展示中心前期工作。加快镇（街道）综合文化站、村级公共文化服务中心、社区文化中心建设。挖掘玉林历史文化资源，组织创作一批文化艺术精品。广泛开展全民健身活动，增强群众体质。

积极发展卫生计生事业。抓好公立医院改革，实现县级公立医院改革全覆盖。完善新型农村合作医疗制度，扩大大病救治保障范围。继续实施国家基本公共卫生服务，加强疾病预防控制，提高群众健康水平。抓好市骨科医院整体搬迁和中心医院、儿童医院等医疗卫生项目建设，推进卫生计生惠民工程。坚持计划生育基本国策，统筹解决人口问题，提高人口素质。

全面推进平安玉林建设。做好“六五”普法工作，完善城乡公共法律服务体系。加强流动人口和特殊人群的服务管理，健全立体化社会治安防控体系，严厉打击各种违法犯罪活动，提升社会公众安全感和满意度。加快信访调处中心建设，健全多元化矛盾纠纷解决机制，依法化解信访积案和历史遗留问题。加强安全管理和应急建设。严格实施社会稳定风险评估机制。

强化安全保障工作。加强食品药品安全检验检测机构建设，完善食品药品安全协调联动机制，强化食品药品过程控制，深入开展专项整治，保障群众食品药品安全。强化安全生产“红线”意识，落实安全生产责任体系，建立安全检查和隐患整治长效机制，夯实安全生产基础。

统筹推进其他各项事业发展。积极支持驻玉部队建设，加强国防动员和“双拥”工作，争创全国双拥模范城。落实民族宗教政策，促进民族团结和宗教和谐。全面实施妇女儿童发展规划，深入开展“三留守”人员关爱行动，保障妇女儿童合法权益。做好老龄工作，加快社会养老服务体系建设。完善残疾人服务和保障体系。加强全民科学素质和道德素质工作。支持各民主党派参政议政。充分发挥工会、共青团、妇联等群团组织的重要作用。进一步加强统计工作。统筹做好外事、侨务、对台事务、人防、气象、防震、水文、测绘、无线电管理、方志、档案等工作。

三、深入建设法治政府

按照中央、自治区和市委关于法治建设的部署，以依法行政为核心，以正风肃纪为抓手，以制度约束为重点，加快建设职能科学、权责法定、执法严明、公开公正、廉洁高效、守法诚信的法治政府。

（一）依法履行政府职能。坚持法定职责必须为、法无授权不可为，实行政府权力清单和责任清单制度，依法规范政府公共服务、市场监管、社会治理和环境保护等职能，推行机构、职能、权限、程序、责任制度法定化，确保行政机关依法行使权力、履行职责。深化行政审批制度改革，推进行政审批流程再造，简化审批工作，提高行政效率。

（二）健全依法决策机制。严格依照法定权限决策，完善行政决策程序，把公众参与、专家论证、风险评估、合法性审查、集体讨论决定确定为重大行政决策的必经法定程序。建立健全行政机关内部重大决策合法性审查机制。

（三）规范行政执法行为。深化行政执法体制改革，推进综合执法，减少执法层级。加强食品药品、安全生产、环境保护等重点领域的基层执法力量，维护公共利益。严格规范公正文明执法，落实行政执法责任制，深入开展行政执法评议考核。

（四）加强行政权力监督。认真执行市人大及其常委会的决议、决定，自觉接受市人大及其常委会的监督，切实做好人大代表建议及政协委员提案办理工作。加强民主监督、行政监督、审计监督、社会监督、舆论监督，确保行政权力公开、透明运行。

（五）全面推进政务公开。深入推行政务公开、执行公开、管理公开、服务公开、结果公开。拓宽政府信息公开渠道，保护公民、法人和其他组织获取政府信息的合法权益。建立行政执法公示制度。建立健全各级政务服务体系，推动政务服务向基层延伸，打通联系服务群众“最后一公里”。

（六）加强廉洁政府建设。持之以恒纠正“四风”，严格控制“三公”经费，倡俭治奢，厉行节约。严格公共资金、公共资源、国有资产监管。健全惩治和预防腐败体系，认真落实“一岗双责”制度，严肃查处违纪违法案件，营造风清气正的发展环境，凝聚全市人民共谋发展、共筑和谐的正能量。

各位代表，在新常态下加快发展、转型发展，需要我们以坚定的决心、全新的思维、有力的举措、务实的作风推进工作。让我们紧密团结在以习近平同志为总书记的党中央周围，在市委的坚强领导下，解放思想、创新进取，勇于担当、攻坚克难，全面开创玉林经济社会发展新局面，为加快建设“两城市一中心”、与全区全国同步全面建成小康社会而努力奋斗！
